# Interstitial Cells of Cajal: Potential Targets for Functional Dyspepsia Treatment Using Medicinal Natural Products

**DOI:** 10.1155/2021/9952691

**Published:** 2021-06-30

**Authors:** Jin-Yong Joung, Seo-Hyung Choi, Chang-Gue Son

**Affiliations:** ^1^Liver and Immunology Research Center, Oriental Medical College of Daejeon University, 75, Daedeok-daero 176 beon-gil, Seo-gu, Daejeon 35235, Republic of Korea; ^2^Department of Internal Medicine, Weedahm Oriental Hospital, 430, Yeoksam-ro, Gangnam-gu, Seoul 06200, Republic of Korea

## Abstract

**Introduction:**

The pathophysiology of functional dyspepsia (FD) remains uncertain, but the interstitial cells of Cajal (ICCs), pacemakers that regulate gastrointestinal motility, are garnering attention as key modulators and therapeutic targets in FD. This review comprehensively discusses the involvement of ICCs in the pharmacologic actions of FD and as therapeutic targets for herbal products for FD.

**Methods:**

A search of the literature was performed using PubMed by pairing “interstitial cells of Cajal” with “medicinal plant, herbal medicine, phytotherapy, flavonoids, or traditional Chinese medicine (TCM).”

**Results:**

From the 55 articles screened in the initial survey, 34 articles met our study criteria. The search results showed that herbal products can directly depolarize ICCs to generate pacemaker potentials and increase the expression of c-kit and stem cell factors, helping to repair ICCs. Under certain pathological conditions, medicinal plants also protect ICCs from oxidative stress and/or inflammation-induced impairment. Two representative herbal decoctions (*Banhasasim-tang,* 半夏泻心汤, and *Yukgunja-tang,* 六君子汤) have been shown to modulate ICC functions by both clinical and preclinical data.

**Conclusion:**

This review strongly indicates the potential of herbal products to target ICCs and suggests that further ICC-based studies would be promising for the development of FD treatment agents.

## 1. Introduction

Functional dyspepsia (FD) is one of the most common gastrointestinal (GI) disorders, affecting 10–30% of the population worldwide [1]. It is characterized as a recurrent or persistent disorder of sensation and movement in the upper digestive tract without any explainable organic causes [2]. Although FD is not generally life threatening, it is known to impair physical, mental, and social aspects of quality of life. The socioeconomic burden of FD was estimated at US$ 18.4 billion in the USA in 2009 [3].

The underlying pathophysiology of FD is not yet fully understood, but *Helicobacter pylori* infection, visceral hypersensitivity, acid disorders, psychosocial factors, and/or abnormal gut motility are considered to be the main contributors to FD [4]. Given these pathological factors, *H. pylori* eradication, proton pump inhibitors (PPIs), histamine-type-2 receptor antagonists, tricyclic antidepressants (TCAs), and prokinetic drugs are widely used for patients with FD [5]. However, these agents have shown relatively low response rates and frequent relapse at rates of up to 73%, which leads to clinical limitations [6]. Therefore, researchers are looking for new targets for the treatment of FD [7].

As key modulators of the pathophysiology of FD, interstitial cells of Cajal (ICCs) are receiving attention. ICCs are a type of interstitial cell found in the GI tract and are known to play a major role in GI motility [8]. ICCs mediate input from the GI motor nervous system to smooth muscle and act as pacemakers in GI motility by generating spontaneous electrical slow waves to stimulate rhythmic peristalsis. Recently, increasing evidence has suggested that loss or dysfunction of ICCs is a cause of GI motility disorders, especially FD [9–11]. Thus, ICCs are currently considered targets for pharmacological intervention for patients with FD.

Herbal products have been used as treatment options for patients with GI disorders. One study reported that 34.7% of surveyed patients with functional GI disorders used herbal medicines [12]. Many researchers are also investigating herbal medicines that can modulate multiple targets via their multiple active components. Most herb-derived benefits for GI disorders are thought to be linked to GI motility [13]. Herbal medicines are also proposed to affect the functions of ICCs, which have complex interactions with surrounding cells and express various receptors for neurotransmitters and circulating hormones [11, 14].

This review aims to analyze the current status and evidence for medicinal natural products related to FD treatment that target ICCs and to increase knowledge regarding ICCs in the context of FD.

## 2. Strategy for Literature Survey and Overall Features of Results

We conducted a literature search using three using PubMed (http://www.ncbi.nlm.nih.gov/pubmed) by pairing “interstitial cells of Cajal” with “herbal medicine, phytotherapy, flavonoids, or traditional Chinese medicine.” The search was conducted on papers published until November 2020.

From the 55 articles screened by the initial survey, a total of 34 related articles were selected, of which 13 described in vivo studies, 18 described in vitro studies, and 3 described both in vivo and in vitro studies. Among those studies, 22 used herbal prescriptions, 4 used herbs, and 8 used flavonoids. Banhasasim-tang (半夏泻心汤), a traditional Chinese medicine, was the most frequently studied compound and was used in 3 studies.

## 3. Overview of ICC Physiology

**Figure 1:**
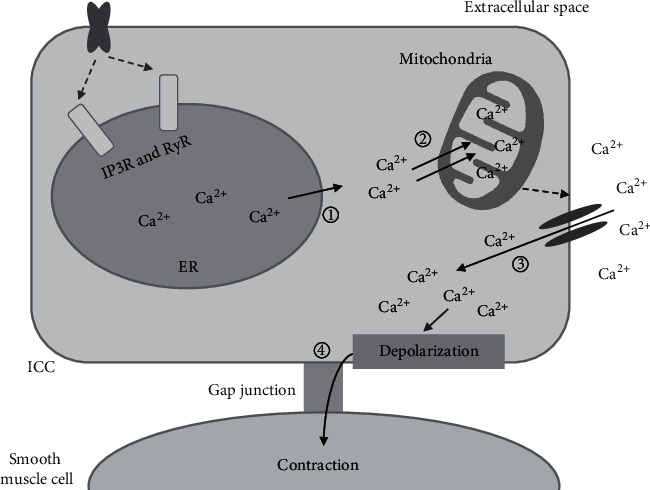
Mechanism for the generation of slow-wave potentials in ICCs. (1) The release of Ca^2+^ into the cytoplasm from the endoplasmic reticulum (ER) is initiated by the activation of ryanodine (RYR) receptors and/or inositol triphosphate 3 (IP3) receptors. (2) Overabsorption of Ca^2+^ into the mitochondria occurs, resulting in a localized drop in Ca^2+^ concentration. (3) Ca^2+^-inhibited nonselective cation ion channels are activated, leading to rapid cellular influx of Ca^2+^ from the extracellular space, which produces spontaneous transient depolarizations (STDs). (4) STDs are delivered to the GI smooth muscles through gap junctions and cause smooth muscle contractions.

ICCs, discovered by Santiago Ramón y Cajal in 1911, are known as end structures of the intrinsic nervous system in the GI tract that mediate nerves and smooth muscle cells [15]. ICCs connect with other ICCs and smooth muscle cells through gap junctions, forming a syncytium in GI tissue [16]. ICCs were first shown to play a key role in GI motility by generating slow waves that induce rhythmic contraction in smooth muscles by Faussone Pellegrini in 1977 [17]. The most widely known mechanism for slow wave generation involves intracellular changes in Ca^2+^ concentrations inside ICCs [11]. In this mechanism, the release of Ca^2+^ into the cytoplasm from the endoplasmic reticulum (ER) is first initiated by the ignition of ryanodine (RYR) receptors and/or inositol triphosphate 3 (IP3) receptors. In response to this, overabsorption of Ca^2+^ into the mitochondria occurs, resulting in a localized decrease in the Ca^2+^ concentration. Next, Ca^2+^-inhibited nonselective cation ion channels are activated, leading to rapid cellular influx of Ca^2+^ from the extracellular space, which is called spontaneous transient depolarization (STD). The STD signal is delivered to the GI smooth muscles through gap junctions and causes smooth muscle contractions (Figure 1). Recently, researchers have found other membrane channels involved in regulating the intracellular Ca^2+^ concentration, including anoctamin 1 (ANO1, a calcium-activated chloride channel) and transient receptor potential melastatin 7 (TRPM 7) [10, 18].

**Figure 2:**
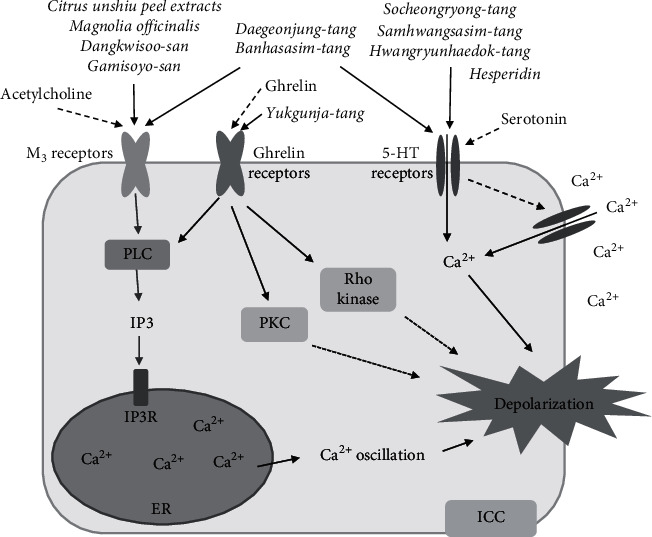
Pathways of the generation of slow-wave potentials by herbal medicines.

The slow-wave potentials in ICCs respond to both neural and nonneural inputs under physiological and/or pathological conditions [19]. ICCs are innervated by myenteric nerves that act as key neural stimulators for GI motility [11]. ICCs act via various receptors for neurotransmitters and circulating hormones. To date, muscarinic acetylcholine receptors (mainly M_3_ receptors), serotonin (5-HT) receptors (mainly 5-HT_3_ and 5-HT_3_ receptors), and G protein-coupled receptors have been the relatively well studied [15, 20, 21] (Figure 2).

M_3_ and M_2_ receptors are important in neuronal signaling from enteric motor neurons [15]. In this process, phospholipase C (PLC) and IP3 act as key molecules for the transformation of signals into ER-derived Ca^2+^ oscillations, leading to depolarization [22]. Serotonergic stimulation, one of the important contributors to the brain-gut connection, also modulates intracellular Ca^2+^ in ICCs and generates slow-wave potentials through 5-HT_3_ and/or 5-HT_4_ receptors. The underlying mechanisms may involve induction of Ca^2+^ influx from the extracellular space through 5-HT receptors and simultaneous activation of voltage-gated Ca^2+^-permeable channels [23]. Ghrelin, known as a hunger hormone, is secreted by enteroendocrine cells and increases food intake. It binds to G protein-coupled receptors and regulates intracellular and extracellular Ca^2+^ concentrations in ICCs, leading to the generation of pacemaker potential via IP_3_-, Rho kinase-, and protein kinase C (PKC)-dependent signaling pathways [24]. In contrast, certain neurotransmitters or hormones can inhibit pacemaker potential in ICCs via nitric oxide/cyclic guanosine monophosphate (NO/cGMP) signaling pathways or noradrenaline-induced stimulation of *β*_1_-adrenoceptors [25, 26].

On the other hand, ICCs also have c-kit, a tyrosine kinase receptor that plays an essential role in the development of ICCs by binding with its ligand, stem cell factor (SCF) [27]. In general, immunohistochemistry of anti-c-kit can be used to examine the distribution and density of ICCs and can also be used to identify the structure of ICCs [28].

## 4. Pathophysiologic Involvement of ICC in FD

Based on the functions of ICCs in generating slow-wave potentials for GI movement, ICC dysfunction can cause many GI motility disorders [29]. FD is known as one of the representative diseases associated with dysfunction of ICCs [30]. Abnormal gastric slow waves have been observed in patients with FD via multichannel electrogastrography [31]. Pathological findings have shown that delayed gastric dysmotility is associated with degeneration of ICCs in both humans [32] and animals [33]. In an FD rat model induced by chronic stress via tail clamping, the levels of autophagic biomarkers in ICCs were increased, whereas the levels of differentiation biomarkers in ICCs, such as c-kit and SCF, were decreased [30]. Although the relationship between ICC dysfunction and FD has not been accurately elucidated, excessive autophagy and abnormal differentiation of ICCs seem to contribute to the pathogenesis of FD [11].

In addition, patients with Crohn's disease [34], ulcerative colitis [35], and chronic bowel obstruction tend to have low ICC counts [36]. Animal studies have reported impaired function of ICCs under proinflammatory conditions [37, 38]. Consistent with these findings, functional recovery or regeneration of ICCs can help ameliorate digestive disorders. In several artificial GI dysmotility rodent models, positive correlations between improvements in ICC structure and GI motility have been found [33, 39, 40]. These data indicate the importance of ICC impairment or dysfunction in pathophysiology and as a therapeutic target, especially in GI motility-associated disorders, including FD.

## 5. Effects of Herbal Products on ICC Receptors and ICC Integrity

Herbal products can directly depolarize ICCs and generate slow-wave potentials. These results have been obtained mainly from cell-based experiments with ICCs using the patch-clamp technique. Many single-herb extracts [41, 42], TCM drugs [14, 26, 43–51], and herb-derived flavonoids [52] depolarize the pacemaker potential of ICCs in a dose-dependent manner. Depending on the herbal products, different receptors and their signaling pathways are involved in this depolarization process. Three receptors, acetylcholine muscarinic M2/M3 receptors, 5-HT_3_/5-HT_4_ receptors, and ghrelin receptors, have been the main focus of the related studies (Table 1 and Figure 2). Compounds containing these herbal products are believed to act as ligands for the receptors above in cell culture conditions.

Herbal medicines are also able to activate c-kit and SCF, which are involved in central pathways in the growth of ICCs. Banhasasim-tang, a typical herbal decoction for patients with FD, has been found to significantly promote ICC and SCF signaling in stomach tissues in both a loperamide-induced FD mouse model and a diabetic dyspepsia rat model [53, 54]. These herbal effects on ICCs and SCF have also been reported in other animal models, including in a trinitrobenzene sulfonic acid-induced rat colitis model treated with Shenqing recipe [40], a cirrhotic ascites rat model treated with Xiaozhang Tie [55], and a loperamide-induced constipation rat model treated with Herba Cistanche [56]. A single flavonoid from citrus fruits and tomatoes, naringenin, has also activated c-kit and SCF, favoring constipation, in a loperamide-induced constipation model [57]. As expected, some herbal drugs (Tong Bian decoction, Jianpi Qingchang decoction, Chaihu Shugan powder, Aurantii Fructus Immaturus, and Atractylodis Macrocephalae Rhizoma) inhibit autophagy of ICCs or alleviate their dysfunction in GI inflammation animal models [58–61]. A summary of the effects of herbal products on ICC integrity is presented in Table 2.

## 6. Effects of Herbal Products against Oxidative Stress-Induced ICC Impairment

Oxidative stress, one of the most common pathological contributors, is also well known to cause both dysfunction and loss of ICCs in the GI tract [62]. Overproduction of NO after surgery has been found to disrupt both GI motility and the slow-wave potentials of ICCs in a mouse model [63]. Cotreatment with interferon-gamma and lipopolysaccharide (IFN-*γ* + LPS) impairs the function and structure of ICCs in tissue culture conditions using the smooth muscle layer of the murine jejunum, while antioxidants and inhibitors of NO synthesis protect against these alterations [64]. In fact, a relatively low concentration of NO, as a neurotransmitter, is essential for ICCs to function properly [65], whereas a higher concentration or long-term exposure to NO can induce ICC damage [66]. The high density of mitochondria in ICCs can explain the increased susceptibility to oxidative stress conditions [67, 68]. As mentioned above, mitochondria are essential for Ca^2+^ handling within ICCs to generate slow waves [69].

On the other hand, many herb-related studies have revealed antioxidant and anti-inflammatory properties that result in protection against or treatment of GI disorders, including FD [70, 71]. Oxidative stress and inflammation coexist in most pathologic conditions [72]. Curcumin, a polyphenol from *Curcuma longa* (turmeric), increases SCF/c-kit protein levels in stomach tissues and ameliorates diabetic gastroparesis in rats by blocking the development of oxidative stress [73]. Quercetin, a plant flavonol, restores the density of ICCs in the jejuna of diabetic rats through its antioxidant action [74]. Disruption of GI motility is one of the complications of diabetic mellitus, and its link to ICCs has also been clearly reported [75]. In addition, FD and diabetic gastroparesis have similar symptoms and share a very similar pathogenesis [76].

ICC dysfunction or a reduction in ICC numbers does not mean permanent loss. ICCs have a high degree of plasticity and can be transformed into a smooth muscle-like phenotype rather than be completely destroyed [77]. Many herb-related studies have shown that ICCs can recover their original phenotype and function when oxidative stress or inflammation is alleviated in a tail clasping stress rat model [33, 78]. These facts suggest that the antioxidant activities of herbal products are key characteristics for reversal of ICC dysfunction under oxidative stress conditions, such as FD.

## 7. Two Representative TCMs Modulating ICC: Banhasasim-Tang (半夏泻心汤) and Yukgunja-Tang (六君子汤)

In clinical practice, the most commonly used TCMs for FD are Banhasasim-tang (半夏泻心汤, known as Ban Xia Xie Xin decoction in China and Hange-shashin-to in Japan) and Yukgunja-tang (六君子汤, known as Liu Jun Zi decoction in China and Rikkunshito in Japan). Due to their widespread use, these two decoctions have been widely studied in both clinical and preclinical studies [79, 80]. Clinical practice guidelines for FD published in Japan recommend the use of Yukgunja-tang as a second-line treatment with evidence level A [81].

Banhasasim-tang has been widely prescribed for patients with FD. Several clinical studies have shown the beneficial effects of BST against gastric dyspepsia [82, 83], and a meta-analysis study reported that Banhasasim-tang is as effective as conventional treatments [84]. Regarding its underlying mechanisms, several preclinical studies have suggested an association with the recovery of ICCs. Banhasasim-tang depolarizes the slow-wave potentials in ICCs by regulating internal and external Ca2^+^ via the M_3_ and 5-HT_3_ receptors in a dose-dependent manner [44]. We also found that Banhasasim-tang can reverse the inactivation of c-kit and modulate oxidative stress and contraction-related genes such as the 5-HT_4_ receptor, ANO1, RYR receptor 3, and smooth muscle myosin light chain kinase in a loperamide-injected mouse model [53]. This decoction also promotes the numbers of stomach ICCs and the levels of SCF in diabetic rats [54].

Yukgunja-tang has also been frequently investigated for its usefulness in FD treatment using both clinical and preclinical studies. Several RCTs have reported the positive effects of Yukgunja-Tang for FD treatment [85, 86]. A meta-analysis analyzed 52 RCTs, and Yukgunja-tang was a strong therapeutic for FD management in patients [87]. The therapeutic mechanisms of Yukgunja-tang are known to involve gastric ghrelin secretion, which promotes ICC depolarization in the GI tract [24]. Yukgunja-tang administration increases the plasma levels of ghrelin in both healthy subjects [88] and FD patients [86]. Yukgunja-tang also acted directly on the ghrelin receptor of ICCs and then depolarized the pacemaker potential in an ICC culture experiment [49]. In addition, the strong antioxidative effects of Yukgunja-tang in the GI tract have been evidenced in animal studies [80, 89], which may contribute to the amelioration of ICC impairment.

## 8. Conclusion

**Figure 3:**
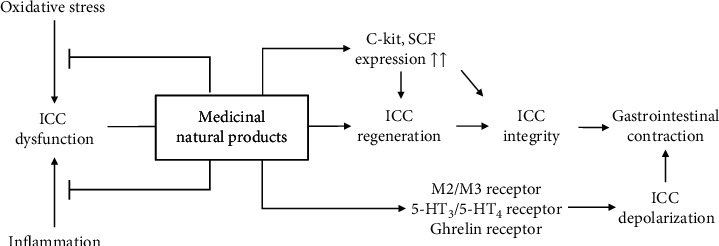
Protective or restorative effects of herbal medicines against ICC dysfunction.

Accumulated evidence has revealed the effects of herbal products on ICCs. These products can increase ICC numbers or restore ICC function. Their therapeutic actions can be categorized as follows: first, they can modulate ICC receptors via ligand-like actions; second, they can protect or restore ICC integrity via activation of c-kit; and third, they can exert antioxidant effects to protect against or reverse ICC dysfunction (Figure 3). The available data focusing on the effects of herbal products on ICCs sufficiently support the importance of ICCs as therapeutic targets of herb-derived treatments for FD. Our study has some limitations, such as a lack of human-derived direct evidence and a lack of identification of active compounds, especially those in decoctions such as Banhasasim-tang and Yukgunja-tang. Nevertheless, our present study suggests that promising new drugs can be developed for FD using herbal resources.

## Figures and Tables

**Table 1:** Medicinal plants acting on the depolarization of ICCs.

Medicinal plants or decoctions (reference)	Molecular mechanism
*Depolarizers of the pacemaker potential of ICCs*	
Gamisoyo-san [14], *Citrus unshiu* peel extracts [41], Dangkwisoo-san [43]	Via M_3_ receptor
*Magnolia officinalis* [42]	Via M_2_ and M_3_ receptors
Banhasasim-tang [44]	Via M_3_ and 5-HT_3_ receptors
Daegeonjung-tang [45]	Via M_3_ and 5-HT_4_ receptors
Hesperidin, a citrus flavonoid [52]	Via 5-HT_4_ receptor
Hwangryunhaedok-tang [46], Samhwangsasim-tang [47], Socheongryong-tang [48]	Via 5-HT_3_ and 5-HT_4_ receptor
Yukgunja-tang [49]	Via ghrelin receptor
Leejung-tang [90], Sengmek-san [50]	Via PLC pathway
Pyungwi-san [51]	Via PKC pathway

*Inhibitors of the pacemaker potential of ICCs*	
Galgeun-tang [26]	Via *α*2- and *β*1-adrenoceptors
Quercetin [91]	Via opioid receptor signaling pathways

M receptors: muscarinic acetylcholine receptors; 5-HT receptors: 5-hydroxytryptamine receptors; PLC: phospholipase C; PKC: protein kinase C.

**Table 2:** Medicinal plants acting on the behaviors of ICCs.

Herbal product	Effect (experimental model)	Molecular mechanism
Jianpi Qingchang decoction [60]	Regulation of intestinal motility of DSS-induced colitis (in vivo)	IL-10, IFN-*γ*, c-kit mRNA ↑↑ TNF-*α*, IL-1, LC3-II, Beclin-1, NF-*κ*B p65 mRNA *β* ↓↓
Banhasasim-Tang [53, 54]	Tempering of loperamide-induced functional dyspepsia (in vivo)	C-kit, nNOS, 5HT4R, ANO1, RYR3 and smMLCK gene ↑↑
	Improvement of gastric motility in rats with diabetes mellitus (in vivo)	ICCs, SCF ↑↑
Tong bian decoction [58]	Enhancement of colon transport function (in vivo)	ICC, c-kit mRNA ↑↑
Shuwei decoction [33]	Improvement of gastric motility in a tail clasping-induced functional dyspepsia rat model (in vivo)	Serum SCF ↑↑, serum NO ↓↓, improvement of the structure of ICCs
Da-Cheng-Qi decoction combined with *Lactobacillus acidophilus* [39]	Improvement of GI motility in mice with traumatic brain injury (in vivo)	Improvement of ICC network structure damage
Shenqing recipe [40]	Repair of the ultrastructure of colonic ICCs in a TNBS-induced colitis rat model (in vivo)	C-kit ↑↑
Chaihu Shugan powder [61]	Inhibition of excessive autophagy of ICCs (in vitro)	Bcl2↑↑, LC3, Beclin-1, PI3KC3 ↓↓
Xiaozhang Tie [55]	Reduction in the degree of ascites and improvement of intestinal motility in cirrhotic rats (in vivo)	SCF, c-kit ↑↑
Herba Cistanche [56]	Improvement of loperamide-induced slow transit constipation (in vivo)	C-kit, SCF, PI3K ↑↑,
Total phenols of *Magnolia officinalis* Rehd. et Wils. [92]	Improvement of gastric motility in an atropine-induced GI dysmotility rat model (in vivo)	SCF, c-kit ↑↑
Aurantii Fructus Immaturus and Atractylodis Macrocephalae Rhizoma [59]	Protection of glutamic acid-stimulated ICCs (in vitro)	Reduction in autophagy via inhibition of the PI3K/Akt/mTOR pathway
Eugenol and cinnamaldehyde (transdermal administration) [93]	Increase in ICC numbers in a trinitrobenzene sulfonate-induced ulcerative colitis rat model (in vivo)	SCF, c-kit ↑↑
Aconitine, emodin [94]	Toxicity toward ICC cells individually but not in combination (aconitine:emodin as 2 : 1) (in vitro)	Deactivation of the Na⁺/K⁺-ATPase pump
Hesperidin, a citrus flavonoid [52]	Increase in GI motility, depolarization of the pacemaker potentials of ICCs (in vivo and in vitro)	Via 5-HT_4_ receptor
Naringenin [57]	Improvement of loperamide-induced constipation (in vivo)	C-Kit, SCF, aquaporin 3 ↑↑
Quercetin [74]	Increase in ICC numbers in a diabetic rat model (in vivo)	(possibly due to its antioxidant action)
Nobiletin [95]	Induction of contraction in weakly contractile states, inhibition of contraction in highly contractile states (in vitro)	Via c-kit-dependent pathway
Eugenol^*∗*^ [96]	Inhibition of intestinal contractions (in vitro)	Inhibition of Ca^2+^-activated Cl^−^ channel TMEM16A in ICCs

^*∗*^Drug inhibiting the function of ICCs.
